# Molecular evolution of *psbA* gene in ferns: unraveling selective pressure and co-evolutionary pattern

**DOI:** 10.1186/1471-2148-12-145

**Published:** 2012-08-16

**Authors:** Lin Sen, Mario Fares, Ying-Juan Su, Ting Wang

**Affiliations:** 1 State Key Laboratory of Virology, College of Life Sciences, Wuhan University, Wuhan, China; 2 CAS Key Laboratory of Plant Germplasm Enhancement and Specialty Agriculture, Wuhan Botanical Garden, Chinese Academy of Sciences, Wuhan, China; 3 Integrative Systems Biology Group, Instituto de Biología Molecular y Celular de Plantas (CSIC-Universidad Politécnica de Valencia (UPV)), Valencia, Spain; 4 Evolutionary Genetics and Bioinformatics Laboratory, Department of Genetics, University of Dublin, Trinity College, Dublin 2, Ireland; 5 State Key Laboratory of Biocontrol, School of Life Sciences, Sun Yat-Sen University, Guangzhou, China

## Abstract

**Background:**

The photosynthetic oxygen-evolving photo system II (PS II) produces almost the entire oxygen in the atmosphere. This unique biochemical system comprises a functional core complex that is encoded by *psbA * and other genes. Unraveling the evolutionary dynamics of this gene is of particular interest owing to its direct role in oxygen production. *psbA * underwent gene duplication in leptosporangiates, in which both copies have been preserved since. Because gene duplication is often followed by the non-fictionalization of one of the copies and its subsequent erosion, preservation of both *psbA * copies pinpoint functional or regulatory specialization events. The aim of this study was to investigate the molecular evolution of *psbA * among fern lineages.

**Results:**

We sequenced *psbA *, which encodes D1 protein in the core complex of PSII, in 20 species representing 8 orders of extant ferns; then we searched for selection and convolution signatures in *psbA * across the 11 fern orders. Collectively, our results indicate that: (1) selective constraints among D1 protein relaxed after the duplication in 4 leptosporangiate orders; (2) a handful positively selected codons were detected within species of single copy *psbA*, but none in duplicated ones; (3) a few sites among D1 protein were involved in co-evolution process which may intimate significant functional/structural communications between them.

**Conclusions:**

The strong competition between ferns and angiosperms for light may have been the main cause for a continuous fixation of adaptive amino acid changes in *psbA *, in particular after its duplication. Alternatively, a single *psbA * copy may have undergone bursts of adaptive changes at the molecular level to overcome angiosperms competition. The strong signature of positive Darwinian selection in a major part of D1 protein is testament to this. At the same time, species own two *psbA * copies hardly have positive selection signals among the D1 protein coding sequences. In this study, eleven co-evolving sites have been detected via different molecules, which may be more important than others.

## Background

The photosynthetic oxygen-evolving photo system II is a unique biochemical system that is capable of oxidizing water molecules [1] and is responsible for producing the almost totality of oxygen on earth [2,3]. *psbA * gene, along with three other chloroplast (cp) genes, namely *psbB *, *psbC * and *psbD *, encodes the core proteins complex in the chlo-roplasts of ferns [4-7]. Precisely, *psbA * gene encodes D1 protein (also known as PsbA protein) in ferns. Physical mapping and pastime sequencing unra-veled a set of genome rearrangements around *psbA* gene in “higher” fern lineages [8-10]. Based on completely and partially sequenced fern pastimes [11-13], the *psbA* gene often locates either to inverted repeat regions (IRs) or to the large single copy region (LSC). Moreover, gene order around it is only found in one state in IRs (*rps7 psbA trnH*), but in two possible arrangements in LSC (*trnK psbA trnH * or *matK psbA trnH*). Impor-tantly, *psbA * gene duplicated when its location shifted from LSC to IRs [13]. This shift mainly affected four fern orders: Schizaeales, Salviniales, Cyatheales and Polypodiales, accounting for over 90% of extant fern diversity. Because both gene copies were preserved since the duplication, we sought to investigate the selective pressures that may drive the evolution of this interesting gene.

Evolution after gene duplication has been a fundamental issue in evolutionary biology chiefly because of its direct link to the generation of novel functions and adaptations [14-16]. The opportunity for generating novel functions is, however, often balanced with the effects such duplications have on gene dosage [17-20]. The photosynthesis environment changed mainly as a result of the emergence of flowering plants [21], and as the open sunny ground transformed into the closed shadowy canopy. For this reason, the biodiversity of other vascular plants largely decreased, and some lineages underwent extinction [22-24]. Remarkably, rather than the result of being adept at holding on in the face of angiosperm domination, the leptosporangiate ferns may have the ability to capitalize upon it [25]. The underlying molecular functioning of this ability remains a mystery for evolutionary biologists, and the present study sheds some light on it. The colonization by these species of canopies angiosperm-dominated light environments sparks the idea that such extraordinary diversification may have been fuelled by functional innovation following *psbA * duplication. To disclose this obscurity, we performed a comprehensive analyze of the action of natural selection following the duplication in *psbA *.

Coevolving sites are non-independent amino acid sites during the course of protein evolution [26,27]. Among the different sites with physical or functional relationships in one protein, one mutation is likely to trigger the corresponding mutations at related sites [28]. Natural selection theory and nearly neutral theory have different explanations for the mechanism of these dynamic changes [29-31]. Despite the debates on the underlying mechanism, the compensatory mutations among the interdependent amino acid sites provide an important approach to understand protein structure and function [32]. The identification of coevolving amino acid sites will not only play a prominent role in the annotations of the function of D1 protein, but will also reflect the evolutionary pattern of the particular protein.

To achieve a better understanding on the evolutionary biology of *psbA * genes in ferns, we focused on three aims: 1) identifying gene order states around *psbA * genes in 11 fern orders; 2) detecting the selective constraints in consequence of *psbA * gene duplication; and 3) unraveling the co-evolution pattern of D1 protein among fern lineages.

## Results

### Sampling and sequencing

**Figure 1:**
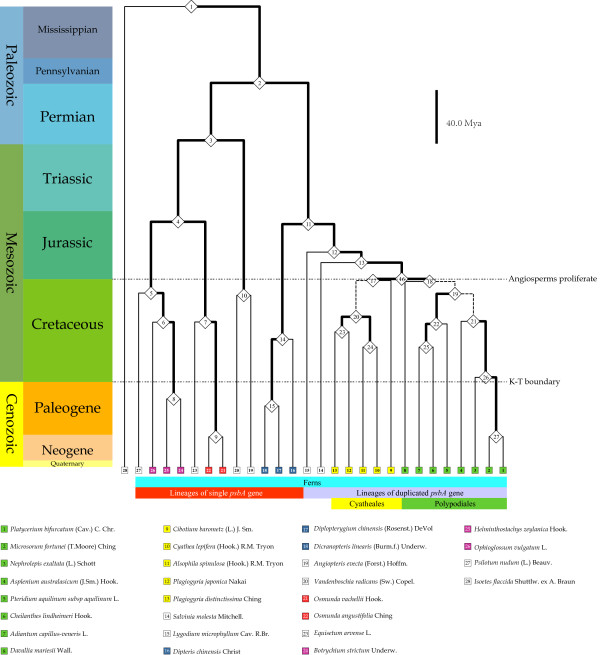
**Detail phylogeny along with geological time-scale of 28 species.** Relied on the combined matrix (dataset 6, Additional file 1 Table S3, Additional file 2), the phylogenetic tree of 28 investigated species was reconstructed via BEAST packages. 27 nodes were numbered and tagged respectively. Length of each branch was in accordance with the estimated divergence time. Estimated parameters of each branch were showed in Table 2. Specific orders mentioned in the text were marked in phylogeny and species names within different orders were illustrated under phylogenies.

**Figure 2:**
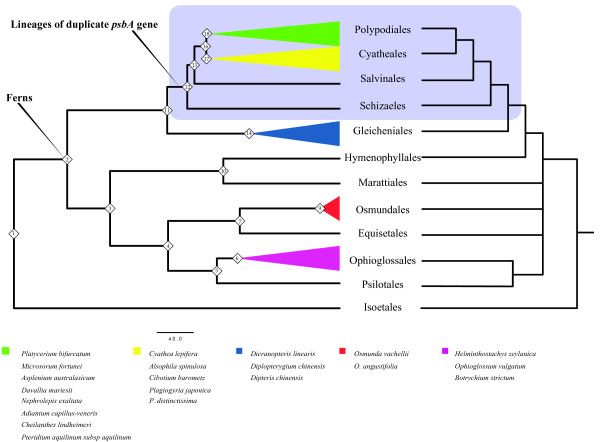
**Summary estimated phylogeny against the accepted fern cladogram.** Left phylogeny was inferred by combined matrix (dataset 6, Additional file 1 Table S3) via UCLD models, which is a summary structure of Figure 1. Right phylogeny is accepted fern cladogram based on several sources [33,34].

Mainly based on Smith’s system [33] and Lehtonen’s recent study [34], we sampled more species in Order Polypodiales (5 species) and Cyatheales (4 species), which have significantly high current fern diversity on earth, and we selected at least one species from the 11 orders (Figures 1, 2, and Table 1). To sum up, these 27 *psbA * sequences represented 11 orders and 14 families (100% of fern orders and 40% of families) providing reasonable coverage of the most taxon-rich lineage. All the sequenced 20 coding regions have 1059nt, coding 353 amino acid residues. Along with their stop code, 20 currently determined *psbA* sequences were uploaded to GenBank (Accession: JQ684679 - JQ684698, Table 1).

**Table 1:** Plant materials and sampling locations in this study

**Order**	**Family**	**Genus**	**Species**	Location*	**GenBank Accession**
Polypodiales	Polypodiaceae	*Platycerium*	*P. bifurcatum* (Cav.) C. Chr.	WBGCAS	JQ684679
		*Microsorum*	*M. fortunei* (T. Moore) Ching	WBGCAS	JQ684680
	Aspleniaceae	*Asplenium*	*A. australasicum* (J.Sm.) Hook*.*	WBGCAS	JQ684681
	Davalliaceae	*Davallia*	*D. mariesii* Wall.	WBGCAS	JQ684682
	Lomariopsidaceae	*Nephrolepis*	*N. exaltata* (L.) Schott	WBGCAS	JQ684683
Cyatheales	Cyatheaceae	*Cyathea*	*C. lepifera* (Hook.) R.M. Tryon	WBGCAS	JQ684684
	Cibotiaceae	*Cibotium*	*C. barometz* (L.) J. Sm.	WBGCAS	JQ684685
	Plagiogyriaceae	*Plagiogyria*	*P. japonica* Nakai	FLBG	JQ684686
		*Plagiogyria*	*P. distinctissima* Ching	WBGCAS	JQ684687
Salviniales	Salviniaceae	*Salvinia*	*S. molesta* Mitchell.	FLBG	JQ684688
Schizaeales	Lygodiaceae	*Lygodium*	*L. microphyllum* Cav. R.Br.	FLBG	JQ684689
Gleicheniales	Gleicheniaceae	*Dicranopteris*	*D. linearis* (Burm.f.) Underw.	FLBG	JQ684690
		*Diplopterygium*	*D. chinensis* (Rosenst.) DeVol	FLBG	JQ684691
		*Dipteris*	*D. chinensis* Christ	WBGCAS	JQ684692
Hymenophyllales	Hymenophyllaceae	*Vandenboschia*	*V. radicans* (Sw.) Copel.	WBGCAS	JQ684693
Osmundales	Osmundaceae	*Osmunda*	*O. vachellii* Hook.	SCBGCAS	JQ684694
		*Osmunda*	*O. angustifolia* Ching	SCBGCAS	JQ684695
Ophioglossales	Ophioglossaceae	*Helminthostachys*	*H. zeylanica* Hook.	SCBGCAS	JQ684696
		*Ophioglossum*	*O. vulgatum* L.	FLBG	JQ684697
		*Botrychium*	*B. strictum* Underw.	WBGCAS	JQ684698

*WBGCAS is short for Wuhan Botanical Garden, Chinese Academy of Sciences, Wuhan, Hubei, China. FLBG stands for Shenzhen Fairy Lake Botanical Garden, Shenzhen, Guangdong,China. SCBGCAS means South China Botanical Garden, Chinese Academy of Sciences, Guangzhou, Guangdong, China.

Since the analysis of molecular adaptive evolution strictly rejects the termination codon (i.e. TAA, TGA, TAG) in a sequence [35], coding sequences in dataset 1, 2 and 3 showed in Additional file 1 Table S3 were applied in the investigation of selective pressures.

### Phylogenetic analysis

The reconstruction of phylogenetic trees is non-sensitive to the stop codes [36-38]; moreover, intergenic sequence of *psbA-trnH* was widely accepted as good indicator for barcode of land plant [39,40]. Coding sequences along with intergenic regions of *psbAtrnH* in dataset 4, 5 and 6 (Additional file 2)were utilized for the reconstruction of the phylogenetic structures for better resolution (Table 2).

**Table 2:** **Selective pressures among*psbA*gene sequences examined via SLAC and FEL models**

Dataset**	**Model**	Numbers and positions of positively/negatively selected site*					
		***p*** **= 0.01**	***p*** **= 0.05**	***p*** **= 0.1**	***p*** **= 0.15**	***p*** **= 0.2**	***p*** **= 0.25**
One	SLAC	0/22	0/78	0/103	0/137	0/141	1(4)/144
	FEL	0/69	1(4)/126	1(4)/150	1(4)/171	1(4)/198	1(4)/210
Two	SLAC	0/4	0/24	0/42	0/71	0/75	0/79
	FEL	0/22	1(155)/56	2(155, 352)/81	3(4, 155, 352)/93	4(4, 155, 350, 352)/120	5(4, 53, 155, 350, 352)/139

*The positions of positively selected codons were showed in brackets. *p* value stands for the statistical significant levels of the models, which is preset before the data manipulation.

** The contexts of the dataset were introduced in **Additional file**1**Table S3.**

In accordance with the phylogenetic structure in pre-vious documents and present analyses (Figure 2), we found that the combined dataset 6 might not be an accurate indicator for the reconstruction of fern phylogenetic structure under geological timescale. Even though the currently gained tree partially coincided with other relevant results from Smith’s system [33] and Lehtonen’s conclusion [34], we could found several disagreements.

Firstly, species from the same Orders were in the same clades (Figure 2): 1) Order Ophioglossales in node 6; 2) Order Osmundales in node 9; 3) Order Gleicheniales in node 14; 4) Order Cyatheales in node 17 and 5) Order Polypodiales in node 18. Secondly, species from the same Families were in the same sub-clade (Figure 1): 1) Family Ophioglossaceae in node 6; 2) Family Osmundaceae in node 9; 3) Family Gleicheniaceae in node 14; 4) Family Plagiogyriaceae in node 23; 5) Family Polypodiaceae in node 27. Thirdly, species from the same genera were together: 1) Genus *Osmunda* in node 9; 2) Genus *Plagiogyria* in node 23. Fourth, most esti-mated node ages had significant posterior probabilities (Table 3).

**Table 3:** The estimated parameters among phylogenetic tree via BEAST software

Node No.*	Estimated Diverge Time**	Posterior probability***	**Geological timescale**	
			**Era**	**Period**
1	359.53	100	Paleozoic	Mississippian
2	299.9	100	Paleozoic	Permian
3	255.03	98.33	Paleozoic	Permian
4	191.39	100	Mesozoic	Jurassic
5	135.7	100	Mesozoic	Cretaceous
6	112.95	100	Mesozoic	Cretaceous
7	113.11	99.05	Mesozoic	Cretaceous
8	53.02	100	Cenozoic	Paleogene
9	23.38	100	Cenozoic	Neogene
10	133.94	99.81	Mesozoic	Cretaceous
11	189.57	93.15	Mesozoic	Jurassic
12	167.73	100	Mesozoic	Jurassic
13	159.5	99.91	Mesozoic	Jurassic
14	99.6	100	Mesozoic	Cretaceous
15	47.54	100	Cenozoic	Paleogene
16	146.76	100	Mesozoic	Cretaceous
17	145.45	100	Mesozoic	Cretaceous
18	144.76	100	Mesozoic	Cretaceous
19	135.12	83.86	Mesozoic	Cretaceous
20	117.2	47.62	Mesozoic	Cretaceous
21	113.65	68.39	Mesozoic	Cretaceous
22	111.6	100	Mesozoic	Cretaceous
23	105.41	100	Mesozoic	Cretaceous
24	93.51	100	Mesozoic	Cretaceous
25	93.48	100	Mesozoic	Cretaceous
26	69.86	100	Cenozoic	Paleogene
27	23.55	100	Cenozoic	Neogene

* As showed in Figure 1 and Figure 2, all the nodes in the phylogenetic tree were numbered and marked.

** Diverge time of each branch was estimated in BEAST software and listed accordingly. The geological time scale (era and period) were inferred from the estimated diverge time of each nodes.

*** Posterior probabilities (PP) of every node were inferred via BEAST. Four of them were lower than 95%: 1) node 11; 2) node 19; 3) node 20; and 4) node 21.

The current phylogenetic tree along with timescale was not accurate according to other published literature [25]. i) Partial structure was not the same with the known phylogeny (Figure 2): the Order Hymenophyllales was near to Marattiales in our study rather than Gleicheniales in well-known tree. ii) Several best estimated node ages were not precise, such as node 21 and 22. iii) Four nodes had low posterior possi-bilities (e.g. node 19, 20, 21 in Table 4).

**Table 4:** **Parameter estimates and log-likelihood values under models of variable*ω*ratios among codons**

**Models**	**Ts/Tv**	***np***	**ℓ**	**Estimated value of parameters**	Positively selected codons**
Dataset one*					
M0: One ratio	4.38	22	-3906.05	*ω* = 0.0184	None
M1a: Near neutral	4.55	23	-3858.84	*p*_0_ = 97.14%, *ω*_0_ = 0.00618; *p*_1_ = 2.86%, *ω*_1_ = 1	Not allowed
M2a: Positive	4.55	25	-3858.83	*p*_0_ = 97.14%, *ω*_0_ = 0.00618; *p*_1_ = 2.86%, *ω*_1_ = 1; *p*_2_ = 0%, *ω*_2_ = 79.26	**91 L (97.2%)**
M3: Discrete	4.60	26	-3854.64	*p*_0_ = 96.31%, *ω*_0_ = 0.00488; *p*_1_ = 3.39%, *ω*_1_ = 0.5646; *p*_2_ = 0.3%, *ω*_2_ = 3.9847	**91 L (99.8%)**
M7: β	4.48	23	-3865.36	*p* = 0.0267, *q* = 0.614	Not allowed
M8: β &ω > 1	4.53	25	-3857.78	*p*_0_ =97.84%, *p* = 0.01575, *q* = 0.46508; *p*_1_ = 2.15%, *ω* = 1.08788	**91 L (99.7%)**
Dataset two*					
M0: One ratio	5.97	29	-3093.72	*ω* = 0.02403	None
M1a: Near neutral	6.07	30	-3061.96	*p*_0_ = 96.57%, *ω*_0_ = 0.00127; *p*_1_ = 3.43%, *ω*_1_ = 1	Not allowed
M2a: Positive	5.80	32	-3061.96	*p*_0_ = 96.57%, *ω*_0_ = 0.00127; *p*_1_ = 3.43%, *ω*_1_ = 1; *p*_2_ = 0, *ω*_2_ = 34.2681	347E (58.3%)
M3: Discrete	5.71	33	-3060.47	*p*_0_ = 46.14%, *ω*_0_ = 0; *p*_1_ = 49.52%, *ω*_1_ = 0; *p*_2_ = 4.31%, *ω*_2_ = 0.61912	None
M7: β	5.75	30	-3064.99	*p* = 0.00981, *q* = 0.19983	Not allowed
M8: β &ω > 1	5.80	32	-3061.93	*p*_0_ =96.61%, *p* = 0.01744, *q* = 2.58197; *p*_1_ = 3.38%, *ω* = 1	347E (81.6%), 349P (61.5%), 353 G (54.4%)
Dataset three*					
M0: One ratio	4.66	47	-5742.02	*ω* = 0.01934	None
M1a: Near neutral	4.87	48	-5654.74	*p*_0_ = 96.39%, *ω*_0_ = 0.00538; *p*_1_ = 3.6%, *ω*_1_ = 1	Not allowed
M2a: Positive	4.18	50	-5656.81	*p*_0_ = 96.47%, *ω*_0_ = 0.0054; *p*_1_ = 3.55%, *ω*_1_ = 1; *p*_2_ = 0, *ω*_2_ = 4.46	71 L (72.4%), 91 L (78.0%)
M3: Discrete	4.90	51	-5637.73	*p*_0_ = 90.54%, *ω*_0_ = 0; *p*_1_ = 8.51%, *ω*_1_ = 0.5308; *p*_2_ = 0.943%, *ω*_2_ = 1.71	**71 L (96.1%)**, **91 L (97.1%)**, 351 V (81.3%)
M7: β	4.78	48	-5648.93	*p* = 0.03078, *q* = 0.63009	Not allowed
M8: β &ω > 1	5.09	50	-5641.45	*p*_0_ =98.21%, *p* = 0.01555, *q* = 0.36594; *p*_1_ = 1.78%, *ω* = 1.19067	**71 L (96.4%)**, **91 L(97.2%)**, 351 V (84.9%)

*The contexts of the dataset were introduced in **Additional file**1**Table S3.** Codons with PP > 95% were in bold. The phylogeny trees applied in the estimation were reconstructed throughout dataset introduced in **Additional file**1**Table S3** accordingly.

**Positions and posterior probability of the positively selected codons were showed in this column. The posterior probabilities were given via Bayes Empirical Bayes (BEB) method.

### Gene order around *psbA * gene in ferns

Results of the PCR amplification indicate that gene order around *psbA * gene in fern species could be mainly classified into three types: *trnK-psbA-trnH *, *matK-psbA-trnH * and *rps7-psbA-trnH *. When universal primers (Additional file 1 Table S1) from conserved sequence of *trnK * and *psbA * were applied in PCR systems, five species (*Botrychium strictum, Ophioglossum vulgatum, Helminthostachys zeylanica, Osmunda angustifolia *and *Osmunda vachellii*) have positive reaction with the right sizes of the fragments. Four species (*Vandenboschia radicans, Dipteris chinensis, Diplopterygium chinensis and Dicranopteris linearis*) showed positive results when the primers were from conserved sequence of *matK *and *psbA *(Additional file 1 Table S2). Other eleven species (*Lygodium microphyllum, Salvinia molesta, Plagiogyria distinctissima, Plagiogyria japonica, Cibotium barometz, Cyathea lepifera, Nephrolepis exaltata, Davallia mariesii, Asplenium australasicum, Microsorum fortune * and *Platycerium bifurcatum*) showed positive results when the primers were from conserved sequence of *rps7 * and *psbA * (Additional file 1 Table S2).

### Selective pressure in different *psbA* genes

As showed in Figures 1 and 2, we tested the action of natural selection in D1 protein using three datasets (Dataset 1, 2 and 3 in Additional file 1 Table S3) comprising both presently determined sequences (20 species in Table 1) and sequences retrieved from GenBank (7 species in Additional file 1 Table S4).

**Figure 3:**
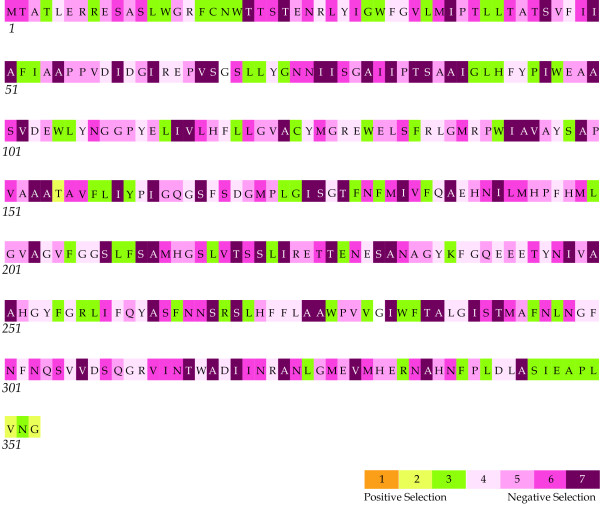
**Selective pressures among 27 fern*psbA*sequences via the MEC model.** Results from Selecton for *psbA* gene were run on 27 fern sequences with the MEC model [42]. The codons were marked according to the sequence from *Platycerium bifurcatum* (JQ684679). Nearly neutral codons were colored in white, negatively selected in magenta, and positively selected in yellow, respectively. Most codons were nearly neutral or under negative selection, only three (155 T, 351 V and 353 G) were positively selected.

We can found subtle positive selection signals in *psbA* genes from fern species (Figure 3, Table 4 and Additional file 1 Table S6). However, when the species were divided into two groups as single and duplicated *psbA* genes, only those from single copy harbored alike signals (Table 4 and Additional file 1 Table S6). As showed in Table 4 and Additional file 1 Table S6, three codons (site 4, 91 and 155) were found as positively selected sites from dataset 1 and none from dataset 2 throughout different mathematics models.

For the detection of positive selection we used nested maximum likelihood models allowing for variation in the ratio of non-synonymous to synonymous substitutions rates (*d*_N_/*d*_S_) across codons, as implemented in PAML and Selecton [41,42], and three models in Datamonkey as well [43,44]. For each dataset, one Likelihood Ratio Test (LRT) was performed for *d*_N_/*d*_S_ heterogeneity across codons (M0/M3 test). The Discrete model (M3) fitted the data significantly better than the one-ratio model (M0), which suggested the existence of significant variation in selective constraints among codons (Table 4, Additional file 1 Table S5). Further, four LRTs were performed for the positively selected codons: M1a/M2a, M7/M8, M8a/M8 and M8a/Mechanistic empirical model (MEC). In order to take the differences between amino-acid replacement rates into account, MEC model employed a cpREV matrix to expand a 20 by 20 amino acid replacement rate matrix into a 61 by 61 sense-codon rate matrix [45]. By this means, a position with radical replacements will obtain a higher Ka value than a position with more moderate replacements (Figure 3). Log-likelihood values and estimates of parameters under various models were given in Table 4, and the likelihood ratio tests in Additional file 1 Table S5. In dataset two, alternative models (M2a and M8), permitting ω > 1, failed to detect positively selected codons and showed non-significance against their null tests (M1a and M7). In M2a, though the estimated value (ω_2_ = 34.2681) is greater than one, no codon (*p*_2_ = 0) belongs to this kind of *ω*; and the estimated value for *ω* was not greater than 1 in M8. Meanwhile, M8 significantly outperformed M7 in both datasets; M2a outperformed M1a as well (Additional file 1 Table S5). Collectively, we can conclude three main points under nested maximum likelihood models (Table 4): 1) subtle positive selection signal was detected in D1 protein from fern species; 2) codons within D1 protein encoded by duplicated *psbA* genes were mainly under negative selection; 3) several codons encoded by single *psbA* gene might have undergone adaptive evolution.

The random-site models, SLAC, FEL, REL and MEC methods were employed to examine the adaptive evolution of the D1 protein in eleven fern orders [41-44]. For dataset one (Table 2 and Additional file 1 Table S6), one positively selected codon (91 L) has been detected via maximum likelihood methods (Table 4), none via SLAC model, one (4 T) via FEL model (*p* = 0.05), two (4 T and 155 T) via REL model (PP > 95%). Our results indicate that 96.99% codons are highly conserved, 2.69% evolve neutrally, and a few (0.31%) are positively selected with *ω* > 1 (Table 4). In the evolution of singleton *psbA* gene, positive selection at a handful of codons has played an important role in the evolutionary dynamics. On the contrary, the majority of codons are under negative selection in dataset two, while a small part is under neutral selection. No positively selected codon was found in the four fern lineages (Order Schizaeales, Salviniales, Cyatheales and Polypodiales). Our results showed that most of the sites are under purifying selection, while a small part is under neutral selection. No sites in the duplicate *psbA* gene were found under positive selection, while 4 have statistical significance in the singleton.

The selective relaxation in duplicated *psbA* genes could be observed in the adaptive selection analyses based on single likelihood ancestor counting (SLAC) and fixed effects likelihood (FEL) methods. Results in Table 2 showed a decrease of selective pressure in duplicated *psbA* genes (Table 2). For instance, 22 sites in dataset 1 were under selection while only 4 in dataset 2 via SLAC (*p* = 0.01). In accordance with FEL, 69 sites in dataset 1 were under selection while only 22 in dataset 2 (*p* = 0.01). Less codons from dataset 2 against dataset 1 were under either positive or negative selection (*p* = 0.01). On the other hand, no codon was identified as positive selection in dataset 2 under REL (Additional file 1 Table S6), while two amino acid sites (4 T and 155 T) were identified in dataset 1.

### Co-evolutionary pattern among D1 protein

Multiple amino acid sites were involved in the co-evolutionary network within D1 protein. CAPS indicated that one co-evolution pair was located between the N-terminal and α helix (Site 4 and 71) [46]. In Datamonkey [43,44], two kinds of methods for detecting co-evolution (one parent and two parents) indicated that other pair has undergone co-evolution (Site 19 and 350). Moreover, the results from InterMap3D [47] indicated that ten pairs have undergone coevolution processes during their evolution dynamics (Table 5).

**Table 5:** Co-evolution pairs with the D1 protein

Software or Website*	Co-evolutionary Pair**	
	**Site1**	**Site2**
CAPS	4	71
Datamonkey***	19	350
InterMap3D	19	350
	53	351
	4	351
	71	92
	4	352
	71	72
	72	281
	4	53
	72	351
	351	353

*Three packages were applied in the current researches [46,47,73].

**Site1, Site 2: Coordinates of each of the interacting residues.

***Two kinds of molecules (two parents and one parent) were applied and gave the same pair.

## Discussion

### Phylogenetic marker

It is a great challenge to develop DNA barcodes for land plants [40,48]. Kress and Erickson (2007) recommended *rbcL* gene and *psbA-trnH* spacer region as universal markers [49]. Our results indicated that the *psbA* and *psbA-trnH* sequences have high resolution at order and family level, but low resolution at several genera level (Figures 1 and 2, Table 5). Further investigation with extra locus will have a deeper reflection on this task.

### Genome rearrangement and *psbA* duplication

The PCR amplification results coincide with the previous conclusion based on the completely and partially sequenced plastomes [10-13]: three kinds of gene order (*trnK-psbA-trnH *, *matK-psbA-trnH * and *rps7-psbA-trnH*) were found in the 20 species from 8 fern orders (Figure 1). The data of the extant complete fern cp genomes showed that the *trnK-psbA-trnH * and *matK-psbA-trnH * fragments are located in the LSC and the *rps7-psbA-trnH * fragment is in the IRs [10,13,50]. Since the large-scale inversion events involving the duplication of *psbA* gene are rare during the evolution history of fern species [8,9], one copy of *psbA* exists in those species of *trnK-psbA-trnH* and *matK-psbA-trnH* types and two in *rps7-psbA-trnH*. Their full-length *psbA* encoding sequences have not been determined in previous studies.

### Evolutionary trajectory and survival strategy

The strong competition between ferns and angiosperms for light may have been the main cause for the evolutionary trajectory of *psbA* gene. Nevertheless, the occurrence of *psbA* duplication provided an alternative survival strategy versus the molecular adaptive evolution at the codons of D1 protein. Several positively selected sites were found in single *psbA* gene copy and none in duplicated *psbA* (Table 2). These results indicated that single *psbA* gene might have functional adaptation via the replacement at certain positions among the D1 protein while the duplicated one might have not. Conversely to the case of single gene copies, the leptosporangiate ferns with duplicate *psbA* gene may have acquired further fitness gain through the existence of a new transcription locus for the synthesis of D1 protein, which might directly increase the efficiency of photosynthesis by protein dosage effects [20,51,52].

The structure and function of the D1 protein are conserved among cyanobacteria, red algae and plants [53,54]. The purifying selection of *psbA* in Lejeuneaceae has been noticed in a recent study [55]. Regretfully, not like the elegant investigation in *rbcL* gene [56], the detail function of the codons in D1 protein remains obscure. Mutation experiments of D1 protein will unveil the functional importance of the positively selected positions. Moreover, the further research on the relationships between positively selected mutations among *psbA* gene from fern species and the biodiversity will have great impacts on the understanding of fern evolutionary biology.

### Complicated intra-molecular evolution under selective pressure

Eleven co-evolving sites (Table 5) have been detected via different molecules, and they (site 4, 19, 53, 71, 72, 92, 281, 350, 351, 352 and 353) may be more important than others in D1 protein. Point mutation experiments may have distinctive outcomes, some could cause severe functional consequences and others could result in completely undetectable change. This fact indicates that a protein is a network of interacting residues, and the core nodes in this network determine the function of the protein [57]. Future point mutation experiments aiming at the eleven sites may have butterfly effect on the protein structure and function.

*psbA* gene, along with three other chloroplast genes, encodes the core proteins complex in the chloroplasts of ferns. However, as we have concluded before the functional adaptation of this complex might be caused by the inter-coevolution among different proteins [32]. The current intra-molecular evolution analysis may have shortcomings on predicting the complicated coevolutionary networks among the entire functional core complex. Further conclusion could not be declared without overall analysis based on the four related genes: *psbA*, *psbB*, *psbC* and *psbD*.

## Conclusion

In the current research we present evidence that point to a complex adaptive process mediating the functional innovation of the D1 protein. This process involves a multiply checking of the structural and functional consequences of the fixation of functionally novel mutations and the amelioration of the effects by such mutations may have through compensatory replacement events. A serial amino acid sites are identified as co-evolution positions while significant positive selection signals are detected in the single copy *psbA* gene from the fern species. One hypothetical scenario is put forward: i) single copy *psbA* gene fern species may adapt to the newly formed living circumstance by the modification of amino acids in D1 protein; ii) by the meantime, the dosage effects of D1 protein are the possible strategy against the rising of angiosperm in *psbA* duplicated ones. The selective relaxation in duplicated psbA genes could be observed throughout different models. However, no evidence stands for the functional divergence in the duplicated *psbA* genes. Future investigation will shed new lights on this question. Although this research covers only a little of the diversity of species in fern, our sampling included all 11 orders. Our research however opens exciting new avenues that will hopefully lead to a more complete understanding of the functional novelties and dosage effects in the D1 protein among ferns.

## Methods

### Sampling of plant materials

Plant materials of 20 fern species for the present investigation were collected from Wuhan Botanical Garden, Fairy Lake Botanical Garden and South China Botanical Garden, respectively (Table 1).

### Isolation of total genomic DNA

For each species, three pieces of fresh leaves were collected to isolate genomic DNA with the modified CTAB protocols [58]. Each sample was dissolved in 50 μl TE buffer. Roughly, the quality was determined by 1% agarose/TAE gel electrophoresis and the quantity was estimated via DNA Ladder (Takara). The bright sample under UV-light with right size was used as the template in PCR reactions. The absorption at 260 and 280 nm of qualified template DNA was measured using a 752 spectrophotometer. The purity and concentration was resolved and calculated by the A_260_/A_280_ ratio and A_260_ absorption value.

### PCR amplification and DNA sequencing

PCR amplification was carried out in 100 μl volumes containing 50 mM KCl, 10 mM Tris–HCl (pH 8.0), 0.1% Triton X-100, 1.5 mM MgCl_2_, 0.2 mM each deoxynucleoside triphosphate, 2 U *Taq* DNA polymerase, 0.3 μM primer, 30 ng genomic DNA and DNA-free water. The 3-step and 2-step PCR protocols employed species-specific and universal primers, respectively. Individually, the annealing temperature in 3-step PCR reaction (Additional file 1 Table S1) of species-specific primers was calculated via Primer 3 (http://frodo.wi.mit.edu/primer3/). The thermo-cycling program was set as: 5 min at 95°C (1 cycle); 45 s at 94°C, 60s at Ta°C, 90s at 72°C (34 cycles); 10 min at 72°C (1 cycle). However, the annealing temperature was ignored in 2-step PCR reaction (Additional file 1 Table S2). The thermo-cycling program was set as: 5 min at 95°C (1 cycle); 60s at 94°C, 150 s at 60°C (35 cycles); 20 min at 72°C (1 cycle). Except as normal reactions, the genomic DNA was excluded from the reaction mix for negative control. Then the molecular weight of PCR products was verified in 1% agarose/TAE gels.

Each qualified DNA fragment amplified by the above steps was recovered and purified with a quick PCR Purification Kit (Promega), and then cloned into PMD19-T vectors (Takara). The plasmids, composed of the vectors and the DNA fragments, were transformed to *Escherichia coli* strain DH5α. Plasmids within positive clones were extracted and sequenced with an ABI PRISM 3730 DNA analyzer. Three clones were sequenced for each amplicon to control *Taq* polymerase errors. The overlapping sequences from various amplification steps were assembled as a single contig. To ascertain the contigs’ locations among cp genomes, the sequencing results were submitted to DOGMA website [59].

### Multiple sequence alignments and best-fit nucleotide substitution model

Six multiple sequence alignments (hereafter MSAs) were established for the present investigation (Additional file 1 Table S3). Nucleotide sequences obtained experimentally (Table 1) plus those retrieved from the public databases (Additional file 1 Table S4) were aligned using the MUSCLE software [60]. Partially fern *psbA* sequences from GenBank were excluded from the present study.

The best-fit nucleotide substitution model for each MSA was selected via jModeltest package [61]. And it was also estimated via the automatic model selection tool at the Datamonkey website (http://www.datamonkey.org) for the coding regions [43,44].

### Reconstruction of phylogeny along with time-scale

*Isoetes flaccida* (GenBank Accession GU191333) was selected as outgroup in the phylogenetic analyses. Two nodes were chosen to constrain for a rate consistent with the known fossils: 1) Since *Osmunda* fossils have been described from the Upper Triassic [62], the Osmundaceae clade was constrained to 199.6 million years ago (Mya, Node 4); 2) According to the fossil *Gleichenia*, node 14 were dated to have been originated 99.6 Mya [63]. Moreover, following previous estimates, another node 21 and 22 were separately dated to 110.5 Mya and 42.5 Mya at the beginning of the running: 3) Pteridaceae clade [64] and 4) Polypodiaceae clade [25].

According to authors’ suggestion [37], to avoid the misspecification of dating and taxon sampling, the empty alignment was run before the real MSAs. Then BEAST 1.7 was allowed to infer topology, branch lengths, and dates for combined datasets [38]. A uniform distribution is applied over the estimating of the absolute ages via the MCMC process. For each MSA, BEAST runs 6 × 10^7^ generations, saving data every 1,000 generations, producing 60,000 estimates of dates under a Yule speciation prior under the uncorrelated lognormal distributed relaxed clock model. Convergence statistics was analyzed in Tracer v 1.5, resulting in 54,000 post-burn-in trees. Before the consensus tree was graphically illustrated by Figtree v.1.3.1, TreeAnnotator v.1.6.1 was utilized to produce maximum clade credibility trees from the post-burn-in trees and to determine the 95% probability density of ages for all nodes in the phylogenetic tree.

### Detection of positively and negatively selected codons

Since identification of positive/negative (non-neutral) evolution is fundamental to the understanding of the process of diversifying/purifying selection, this subject has been the focus of several decades of mathematical and computational efforts. Different scientists have developed numerous analysis models and methods, and each has its own advantages [42-44,65]. However, the general consensus of them is that non-synonymous nucleotide substitutions (*d*_N_), whose alternatives leading to a change in the codon and its corresponding amino acid, can be time-scaled by the number of synonymous replacements (*d*_S_), which are nucleotide changes that only change the codon but not the amino acid and are consequently neutrally fixed and proportional to the divergence time between the sequences.

The random-site models (M0 vs. M3, M1a vs. M2a, M5 vs. null test, M7 vs. M8, and MEC vs. null test), contained in PAML package version 4.1 and Selecton version 2.2, allow the ω ratio (ω = *d*_N_/*d*_S_) to shift among codons within the MSA and this parameter is estimated by maximum-likelihood value via Bayesian inference approach [41,42]. Additionally, the results from Selecton version 2.2 are visualized with seven-color scale for representing the different types of selection. To identify the statistical significant levels of the results, the LRT was conducted to compare the nested models [66].

Besides, another three models for detecting codons under selection are implemented on Datamonkey website [43]: SLAC, FEL and REL. SLAC originated from the Suzuki–Gojobori counting approach [67] and is quite efficient on detecting non-neutral evolution in large MSAs. Less conservative than SLAC, FEL fits a site-specific *d*_N_ and *d*_S_ in the context of codon substitution models and tests whether *d*_N_ = *d*_S_, outperforming other two models in MSAs of intermediate size. As the most powerful model of the three, REL is improved based on the Nielsen–Yang approach [68]. Before the analysis of Natural selection, the best-fit nucleotide substitution models in the MSAs were calculated via the model-selection molecule online. Different from the posterior LRTs in the nested models, the parameter for statistical significant level (*p* value or Bayesian factor) was pre-set prior to the estimating processes [69].

### Analysis of inter-dependent evolutionary sites

To understand the broad implications of the amino acid replacements in D1 proteins from fern species, the analysis of the evolutionary dependencies among sites to identify functional/structural dependencies among residues were conducted via five.

Based on a tree-ignorant strategy, CAPS outperforms the tree-aware strategy methods as reported by previous published work [70], which compares the correlated variance of the evolutionary rates at 2 sites corrected by the time since the divergence of the 2 sequences [46]. The significance of the results was evaluated by randomization of pairs in the alignment, calculation of their correlation values, and comparison of the real values with the distribution of 10,000 randomly sampled values. The step-down permutation procedure was employed to correct for multiple tests and non-independence of data [71]. An alpha value of 0.001 was applied to minimize Type I errors. The correlated variability between amino acid sites was weighted by the level of substitutions per synonymous site in order to normalize parameters by the time of sequence divergence [72].

The mathematical models for detecting the co-evolving residues in protein from InterMap3D and Datamonkey websites are based on a tree-aware strategy [47,73]. Currently, three different models, namely Row and Column Weighing of Mutual Information (RCW-MI), Dependency and Mutual Information/Entropy (MI/E), were implemented at the former website [47], and one (Spidermonkey) at the later [43,73]. RCW-MI and Dependency extract both the entropy dependency and the phylogenetic signal [74,75], while the MI/E extracts the entropy dependency from the signal by dividing mutual information by the joint site’s entropy [76,77]. Spidermonkey molecule reconstructs the substitution history of the MSAs by ML-based methods, and analyses the joint distribution of substitution events by Bayesian graphical models [73].

## Abbreviations

cp: Chloroplast; FEL: Fixed effects likelihood; IR(s): Inverted repeat region(s); LSC: Large single copy region; LRT: Likelihood ratio test; Mya: Million (1 × 10^6^) years ago; MI/E: Mutual information/entropy; MSA(s): Multiple sequence alignment(s); PS II: Photo system II; REL: Random effects likelihood; RCW-MI: Row and column weighing of mutual information; SLAC: Single likelihood ancestor counting; Ta: Annealing temperature.

## Competing interests

The authors declare that they have no competing interests.

## Authors' contributions

LS carried out the experimental works, participated in the sequence alignment, molecular evolution analysis and drafted the manuscript. MF contributed to the analysis tools and drafted the manuscript. TW and YJS conceived of the study, and participated in its design and coordination. All authors read and approved the final manuscript.

## Supplementary Material

Additional file 1 **Table S1.**Universal primers for fern psbA gene. S2 Species-specific primers for psbA gene from eight fern species. S3 Six datasets of different species and fragments. S4 Species and accession number of the retrieved data. S5 LRTs of the random-site models in PAML version 4.1*. S6 Positively selected codons determined via REL model Click here for file

Additional file 2 **Currently determined 20 sequences.**The sequences started at the ATG codon of *psbA,* ended before the ATG codon of *trnH.* They included the stop codon of psbA and the intergenic region between psbA and *trnH.* They were applied to reconstruct the phylogenetic trees. Click here for file
