# Supplementary material for: Molecular evolution of psbA gene in ferns: unraveling selective pressure and co-evolutionary pattern
Source: BMC Evol Biol. 2012 Aug 16;12:145. doi: 10.1186/1471-2148-12-145 (PMC3499216; doi:10.1186/1471-2148-12-145)
Supplement: Additional file 1 — Table S1.Universal primers for fern psbA gene. S2 Species-specific primers for psbA gene from eight fern species. S3 Six datasets of different species and fragments. S4 Species and accession number of the retrieved data. S5 LRTs of the random-site models in PAML version 4.1*. S6 Positively selected codons determined via REL model [file 1471-2148-12-145-S1.doc]

# Supplementary tables and captions

**Table S1 Universal primers for fern *psbA* gene**

| Primer name^**^ | Location | Primer Sequence (5'-3') | | Ta ˚C  (Annealing temperature) |
| --- | --- | --- | --- | --- |
| P101 | *rps7* | F: | TGGGAGTGCCATACGGAAGAA | 54 |
|  | *psbA* | R: | GTGCTCAGCCTGGAATACGAT |  |
| M101 | *trnK* | F: | AGTTCCGGGTTCGACCCCCG | 52 |
|  | *psbA* | R: | AGCAGCTGCAACAGGAGCTG |  |
| H101 | *psbA* | F: | GTTATGCATGAACGTAATGCTC | 53 |
|  | *trnH* | R: | CGCGCATGGTGGATTCACAATCC |  |
| Z101 | *rps7-psbA* | F: | AAGCTTACTGGCCCGGGGAATCA | 55 |
|  | *psbA-trnH* | R: | GGGGTAGGAGTTTACAAACATTCCGCA |  |
| J101 | *trnK-psbA* | F: | ATCGGATGGTTTGGTGTCTTG | 56 |
|  | *psbA-trnH* | R: | ATGATGTCAGCCCAGGTGTTT |  |
| N101 | *matK* | F: | TCGATTGTAACCGGGAAAATCGTTTCC | 53 |
|  | *psbA* | R: | GGTCGTGTAATCAACACCTGGGCTG |  |

* Universal primers were applied in 2-step PCR reaction ignoring the annealing temperature. However, the length of amplification products was estimated according to the extant data in GenBank (NC_004766, NC_012818, NC_003386, NC_008829 and HM021798).

** Primers P101F/P101R, M101F/R, H101F/R, Z101F/R, J101F/R and N101F/R could be applied in eleven, nine, twenty, eight, four and three species, respectively.

**Table S2** Species-specific primers for *psbA* gene from eight fern species

| Species | Primer Sequence (5'-3') | | Ta(˚C)* | Length** |
| --- | --- | --- | --- | --- |
| *Cyathea lepifera* | F: | TAGCTGGCGTATTCGGCGGC | 53 | 556bp |
|  | R: | TCCGTTCTACCGCACGAACCT |  |  |
| *Nephrolepis exaltata* | F: | ACATCGTAGCCGCTCACGGC | 54 | 412bp |
|  | R: | TGCCGCACGAACCTTCATCTCA |  |  |
| *Lygodium scandens* | F: | GGCGTATTCGGCGGCTCTCT | 53 | 594bp |
|  | R: | CATCGCGTGGAGTCACGGCA |  |  |
| *Dicranopteris linearis* | F: | AGGCATCTGGTTCACCGCCT | 53 | 489bp |
|  | R: | CGGGATGTGTTCGAGGTGACACA |  |  |
| *Dipteris chinensis* | F: | GCCGCTTGGCCCGTAGTAGG | 54 | 641bp |
|  | R: | CCACTTGGCTACGTCCGCCC |  |  |
| *Diplopterygium chinensis* | F: | CTGGCGTATTCGGCGGCTCT | 55 | 797bp |
|  | R: | TGGGGTCATTCAAGCGGTGC |  |  |
| *Vandenboschia radicans* | F: | TGGCGTATTCGGCGGCTCTC | 55 | 898bp |
|  | R: | TGGCTACGTCCGCCCTCTCT |  |  |
| *Helminthostachys zeylanica* | F: | TGCTACATGGGTCGTGAGTGGGA | 54 | 963bp |
|  | R: | TCGGAAAGGGCAAGGGATGCA |  |  |

* Annealing temperature (Ta ˚C) was calculated via Primer 3 (http://frodo.wi.mit.edu/primer3/).

**Length of the products were verified by the multiply sequencing results.

**Table S3** Six datasets of different species and fragments

| No. | Species | Fragments |
| --- | --- | --- |
| 1 | *psbA* gene in LSC region | Full-length coding region without stop codon** |
| 2 | *psbA* gene in IR regions |
| 3 | All species* |
| 4 | *psbA* gene in LSC region | *psbA* encoding sequence and *psbA-trnH* intergenic regions*** |
| 5 | *psbA* gene in IR regions |
| 6 | All species* |

*Twenty-seven species from Table 1 and Table S4 were included in this dataset 6.

**Since it was strictly rejected in the molecular adaptive evolution models, the stop codon was excluded in the encoding region alignments. Specifically, dataset 1, 2 and 3 were utilized in the selective pressure analysis via different models harboured within PAML package, Selecton and Datamonkey websites.

***To obtain a better phylogeny with estimated time-scale, the combined datasets (No. 4, 5 and 6) were applied via BEAST based on a relaxed molecular clock. Consequently, the results were utilized in the analysis of the adaptive evolution respectively as the guide phylogeny structures.

**Table S4** Species and accession number of the retrieved data

| Order | Family | Genus | Species | GenBank Accession |
| --- | --- | --- | --- | --- |
| Polypodiales | Dennstaedtiaceae | *Pteridium* | *P. aquilinum subsp aquilinum* L. | NC_014348 |
|  | Pteridaceae | *Cheilanthes* | *C. lindheimeri* Hook. | NC_014592 |
|  |  | *Adiantum* | *A. capillus-veneris* L. | NC_004766 |
| Cyatheales | Cyatheaceae | *Alsophila* | *A. spinulosa* (Hook.) R.M.Tryon | NC_012818 |
| Equisetales | Equisetaceae | *Equisetum* | *E. arvense* L. | GU191334 |
| Marattiales | Marattiaceae | *Angiopteris* | *A. evecta* (Forst.) Hoffm. | NC_008829 |
| Psilotales | Psilotaceae | *Psilotum* | *P. nudum* (L.) Beauv. | NC_003386 |
| Isoetales | Isoetaceae | *Isoetes* | *I. flaccida* Shuttlw. ex A. Braun | GU191333 |

*Though several other partial plastomes of fern species have already been published , it is the encoding regions of *psb*A gene that were not fully obtained. To avoid multiply gaps in alignments, the partial encoding sequence data of D1 protein from those species were excluded from present investigation.

**Table S5** LRTs of the random-site models in PAML version 4.1*

| Dataset** | Model | 2△**ℓ** | *p*-value | α=0.05 | α=0.01 |
| --- | --- | --- | --- | --- | --- |
| One | M0/M3 | 102.82 | 2.4×10^-21^ | ● | ● |
|  | M1a/M2a | 0 | Non-significant | ○ | ○ |
|  | M7/M8 | 15.16 | 5.1×10^-4^ | ● | ● |
|  | M8a/M8 | 15.62 | 9.8×10^-5^ | ● | ● |
| Two | M0/M3 | 66.51 | 1.24×10^-13^ | ● | ● |
|  | M1a/M2a | 0 | Non-significant | ○ | ○ |
|  | M7/M8 | 6.17 | 0.0472 | ● | ○ |
|  | M8a/M8 | 6.14 | 0.0135 | ● | ○ |
| Three | M0/M3 | 208.58 | 5.37×10^-44^ | ● | ● |
|  | M1a/M2a | 0 | Non-significant | ○ | ○ |
|  | M7/M8 | 14.96 | 5.6×10^-4^ | ● | ● |
|  | M8a/M8 | 14.82 | 1.1×10^-4^ | ● | ● |

*●Stands for significant result and ○ for non-significant.

**The contexts of the datasets were introduced in Supplementary Table 3.

**Table S6** Positively selected codons determined via REL model

| Dataset | Positively selected codon | | *d*_S_ | *d*_N_ | Posterior Possibility |
| --- | --- | --- | --- | --- | --- |
|  | No. | Position |  |  |  |
| One | 1 | 4* | 0.1769 | 0.4208 | 97.05% |
|  | 2 | 155* | 0.2002 | 0.4278 | 96.79% |
| Two | 1 | 53 | 0.2693 | 0.7535 | 93.57% |
|  | 2 | 155 | 0.2685 | 0.7559 | 93.94% |
|  | 3 | 348 | 0.6304 | 0.7559 | 62.95% |
|  | 4 | 350 | 0.2852 | 0.7559 | 92.51% |
|  | 5 | 351 | 0.5726 | 0.7559 | 67.90% |
|  | 6 | 352 | 0.2778 | 0.7559 | 93.15% |
| Three | 1 | 4* | 0.3459 | 0.4641 | 98.88% |
|  | 2 | 53 | 0.4428 | 0.4640 | 91.27% |
|  | 3 | 155 | 0.4415 | 0.4641 | 91.38% |
|  | 4 | 346 | 0.5787 | 0.4589 | 79.73% |
|  | 5 | 348 | 0.3915 | 0.4560 | 93.63% |
|  | 6 | 349 | 0.4931 | 0.4602 | 86.68% |
|  | 7 | 350 | 0.5152 | 0.4640 | 85.60% |
|  | 8 | 352 | 0.5181 | 0.4640 | 85.36% |

*PP>95%
